# Expanding the Scope of Open Weighing to Target Anxiety About the Consequences of Weight Gain in Cognitive‐Behavioral Therapy for Eating Disorders

**DOI:** 10.1002/eat.24571

**Published:** 2025-10-16

**Authors:** Jamal H. Essayli, Kaitlin A. Hill, Hana F. Zickgraf, Lauren N. Forrest, Cheri A. Levinson

**Affiliations:** ^1^ Penn State College of Medicine Department of Pediatrics, Department of Psychiatry & Behavioral Health Hershey Pennsylvania USA; ^2^ Clinical Psychologist, OCD Institute of Texas Houston Texas USA; ^3^ Research Psychologist, Rogers Behavioral Health Oconomowoc Wisconsin USA; ^4^ University of Oregon Department of Psychology Eugene Oregon USA; ^5^ University of Louisville Department of Psychological and Brain Sciences, Department of Pediatrics, Division of Child and Adolescent Psychiatry and Psychology Louisville Kentucky USA

**Keywords:** anorexia nervosa, anxiety, bulimia nervosa, cognitive‐behavioral therapy (CBT), eating disorders, open weighing, weight, weight gain

## Abstract

**Objective:**

Anxiety about weight gain is a central feature of eating disorders (EDs) and plays a key role in maintaining ED symptomatology. Cognitive‐behavioral therapy (CBT) experts have observed that patients with EDs often believe regular eating will lead to immediate, dramatic, and/or uncontrollable weight gain, prompting interventions like “collaborative weighing” and “open weighing” that help patients develop a realistic understanding of eating‐weight relationships. However, patients also report anxiety about the *consequences* of weight gain (e.g., social rejection, self‐hatred, permanent body dissatisfaction). While various CBT interventions address concerns about the consequences of weight gain, we propose that open weighing sessions—when patients confront weight increases and process accompanying emotional responses—provide underutilized therapeutic opportunities to work with these beliefs in real‐time.

**Method:**

This paper outlines three clinical enhancements that systematically leverage open weighing sessions to address consequence‐related beliefs: (1) identifying catastrophic beliefs about weight gain consequences through guided inquiry, (2) integrating cognitive restructuring by framing weight restoration as a behavioral experiment, and (3) combining in vivo weighing with imaginal exposure to feared weight gain consequences.

**Results:**

These enhancements may provide complementary techniques for capitalizing on patients' real‐time emotional activation during weighing, potentially enhancing existing CBT protocols' effectiveness in addressing a key mechanism underlying restrictive EDs.

**Discussion:**

Future research might include randomized controlled trials comparing various weighing approaches, mixed‐methods evaluations exploring weight‐related fears across ED diagnoses and weight status, and mechanistic studies examining how addressing consequence‐related beliefs improves treatment outcomes.

**Summary**

- People with eating disorders experience anxiety about the consequences of weight gain, such as social rejection, self‐hatred, and permanent body dissatisfaction.
- While existing cognitive‐behavioral therapy protocols address these concerns throughout treatment, open weighing sessions may provide underutilized opportunities to work with these beliefs in real‐time, especially when patients experience weight increases.
- This paper proposes three enhancements to open weighing that leverage patients’ real‐time emotional responses: identifying catastrophic beliefs through guided inquiry, integrating cognitive restructuring that frames weight restoration as a behavioral experiment, and combining in vivo weighing with imaginal exposure to feared consequences.

## 1 Introduction

Anxiety and fear about weight gain are central features of eating disorders (EDs), particularly anorexia nervosa, where they serve as diagnostic criteria (American Psychiatric Association 2013). While anxiety and fear are related emotions, they differ in temporal orientation: fear involves present‐oriented responses to immediate threats, whereas anxiety involves future‐oriented apprehension (Sylvers et al. 2011). Weight gain concerns likely involve both immediate fear responses (e.g., distress seeing higher numbers on a scale) and future‐oriented anxiety about longer‐term consequences (e.g., social rejection). While we acknowledge that both anxiety and fear contribute to weight gain concerns in EDs, we generally frame our approach around anxiety‐based processes, as patients' concerns about the consequences of weight gain are largely future‐oriented (Murray, Treanor, et al. 2016). Weight‐related fear and anxiety extend across ED diagnoses (Brown and Levinson 2022; Butler and Heimberg 2023; Stice et al. 2021; Trompeter et al. 2019), and predict decreased calorie consumption (Steinglass et al. 2010), lower recovery rates (Zerwas et al. 2013), and higher relapse rates (Yackobovitch‐Gavan et al. 2009). Given evidence that anxiety about weight gain maintains ED symptomatology (Murray et al. 2018), developing treatments that effectively target anxiety could improve outcomes.

## 2 Translating Anxious Beliefs to Treatment Development

Clinical experience and growing research suggest that patients with EDs vary in their specific weight‐related concerns (Brown and Levinson 2022; Butler and Heimberg 2023; Levinson et al. 2019). Cognitive‐behavioral therapy (CBT) experts have observed that patients with EDs often believe that regular eating and abstinence from compensatory behaviors (e.g., vomiting) will lead to immediate, dramatic, and/or uncontrollable weight gain, prompting the development of interventions to address these beliefs. For example, Fairburn's CBT—Enhanced (CBT‐E) includes “collaborative weighing,” where therapists support patients in viewing their weight weekly and plotting it on a graph. Among other aims, collaborative weighing helps patients develop a more realistic understanding of eating‐weight relationships (Fairburn 2008). In Waller and colleagues' exposure‐oriented “open weighing” approach, patients predict their weight before stepping on the scale to maximize discrepancies between predicted and actual weights (Becker et al. 2019; Waller et al. 2007, 2019; Waller and Mountford 2015). This helps patients learn that eating is far less likely to cause the dramatic, rapid, or uncontrollable weight gain they fear.

In addition to concerns about how eating affects weight, individuals with EDs report anxiety about the *consequences* of weight gain (Brown and Levinson 2022; Butler et al. 2023; Levinson et al. 2019). These feared consequences vary across patients and might include beliefs that weight gain will result in social rejection, self‐hatred, permanent body dissatisfaction, loss of self‐control, or a meaningless life (see Table 1). Several existing CBT interventions address feared consequences of weight gain. CBT protocols include education about the psychological effects of undereating, developing a joint clinical formulation, and motivational strategies such as exploring the pros and cons of recovery, all of which can help patients question their beliefs about the consequences of both maintaining and changing their weight status (Fairburn 2008; Garner et al. 1997; Vitousek et al. 1998; Waller et al. 2007). Open weighing is likely to enhance weight tolerance and challenge beliefs that seeing one's weight will result in loss of control or increased ED symptoms (Becker et al. 2019; Waller et al. 2007, 2019). CBT‐E contains modules that address the over‐evaluation of weight and shape and low self‐esteem (Fairburn 2008), which include discussion of weight‐related consequences. Body image interventions, such as mirror exposure (Butler and Heimberg 2020) and body neutrality approaches (Mancin et al. 2024), may work in part by challenging body‐related beliefs, potentially reducing patients' fears about body dissatisfaction as a consequence of weight gain.

**TABLE 1:** Frequently reported feared consequences of weight gain by individuals with eating disorders.

Feared consequences of weight gain
I will be judged, rejected, and/or abandoned by everyone in my life I will look ugly, hideous, and/or disgusting I will become paralyzed by weight‐related thoughts, emotions, and/or physical sensations I will become so distracted by my body that I won't be able to focus on anything else I will permanently hate my body I will lose control of my life My life will become meaningless and/or amount to nothing I will live a lonely life I will be unlovable I will permanently hate myself I will become permanently unhappy, sad, and/or depressed I will become permanently anxious, worried, and/or afraid I will no longer have value and/or worth I will no longer have confidence and/or a sense of self My life will be worse off

While existing CBT protocols address feared consequences of weight gain throughout treatment, we propose that open weighing sessions—when patients confront weight increases and process the accompanying emotional and cognitive responses—provide underutilized therapeutic opportunities. We posit that anxiety about the consequences of weight gain is a key mechanism underlying restrictive EDs (Levinson et al. 2014; McEntee et al. 2023; Murray, Loeb, and Le Grange 2016). Theoretically, once patients stop viewing weight gain as catastrophic, downstream fears about eating's impact on weight should diminish accordingly. We therefore offer recommendations for enhancing open weighing within existing CBT frameworks by systematically working with patients' real‐time reactions—especially when they confront weight increases—through focused cognitive and exposure interventions that aim to address catastrophic beliefs about the consequences of weight gain.

## 3 Expanding the Scope of Open Weighing

We describe three clinical recommendations for expanding open weighing to address anxious beliefs about the consequences of weight gain. These should be considered within the context of ongoing debates about weighing practices. Evidence‐based protocols themselves differ, with CBT‐E's collaborative weighing (Fairburn 2008) contrasting with Waller and colleagues' exposure‐oriented method (Fairburn 2008; Waller et al. 2007; Waller and Mountford 2015). Additionally, many patients and approximately half of clinicians prefer “blind weighing,” where weight information is concealed, due to concerns that open weighing may impede recovery (Forbush et al. 2015; Froreich et al. 2020; Wagner et al. 2022). While one study found no significant differences between open and blind weighing on the rate of weight gain or length of stay (Shear et al. 2023), the highly monitored inpatient setting may have constrained patients' behavioral responses (e.g., restriction), potentially masking differences between weighing approaches. Optimal weighing practices remain a significant knowledge gap.

### 3.1 Using Open Weighing to Identify Anxious Beliefs About the Consequences of Weight Gain

Individuals with EDs often experience intense emotional reactions when seeing weight increases. We propose that these reactions provide valuable opportunities to identify and modify weight‐related beliefs. Before beginning open weighing, clinicians could provide psychoeducation about the CBT model (Tolin 2024) and frame open weighing as a tool for accessing weight‐related cognitions and emotions. Especially for patients in higher‐weight bodies, therapists may consider providing psychoeducation about systemic weight bias, anti‐fat attitudes, society's thin ideal, and how these factors shape beliefs about weight gain (Levinson et al. 2024; Pearl et al. 2020; Puhl and Suh 2015; Stice et al. 2013).

When patients experience weight increases, therapists can guide them in identifying their emotional responses and underlying thoughts. This might begin with validating that patients' emotional reactions (e.g., anxiety, sadness) are reasonable given their beliefs about the consequences of weight gain (e.g., “my life will become meaningless”), and that these beliefs are understandable within a society perpetuating the message that “weight gain is bad” (Brown et al. 2022). Indeed, a challenge in treating EDs is distinguishing between irrational and valid beliefs about weight gain. Many ED‐related beliefs are grounded in reality: weight loss can be egosyntonic and externally reinforced (Essayli and Vitousek 2020; Garner and Bemis 1982; Haynos et al. 2021; Vitousek et al. 1998; Vitousek and Hollon 1990; Walsh 2013), individuals in higher‐weight bodies face stigma (Puhl and Suh 2015), and weight gain often leads to initial increases in body dissatisfaction (Bachner‐Melman et al. 2006; Keys et al. 1950). Therapists should acknowledge these valid concerns rather than attempting to restructure realistic beliefs.

Instead, clinicians might focus on identifying catastrophic beliefs about the consequences of weight gain that are amenable to disconfirmation. While temporarily feeling worse about one's body during weight restoration is expected, the belief “I will permanently hate my body if I gain weight” represents an outcome that is both genuinely awful and unlikely. Similarly, while patients in higher‐weight bodies may experience increased judgment, this does not mean they “will no longer have worth” or their lives “will become meaningless.” Anti‐fat stigma negatively impacts mental health (Rubino et al. 2020), yet individuals in higher‐weight bodies can achieve increased self‐confidence and life satisfaction (Godoy‐Izquierdo et al. 2020) after gaining weight in ED recovery. To uncover catastrophic beliefs about the consequences of weight gain, clinicians can use the ED Fear Questionnaire (Levinson et al. 2019), a list of common weight‐related fears (see Table 1), the “downward arrow” technique (Tolin 2024), or similar methods for identifying “worst case scenarios.”

These long‐term consequences build upon the shorter‐term beliefs addressed in some protocols, including beliefs about the degree of weight gain and fears that seeing one's weight will cause loss of control or increased symptoms (Becker et al. 2019; Waller et al. 2007, 2019; Waller and Mountford 2015). For example, the belief “If I gain weight, I will have to vomit” likely stems from an underlying assumption that weight gain is bad and must be counteracted. Addressing beliefs about the consequences of weight gain (i.e., *why* weight gain is bad) may help “break” the connection between “weight gain” and “I should vomit.”

### 3.2 Integrating Open Weighing With Cognitive Restructuring

**FIGURE 1:**
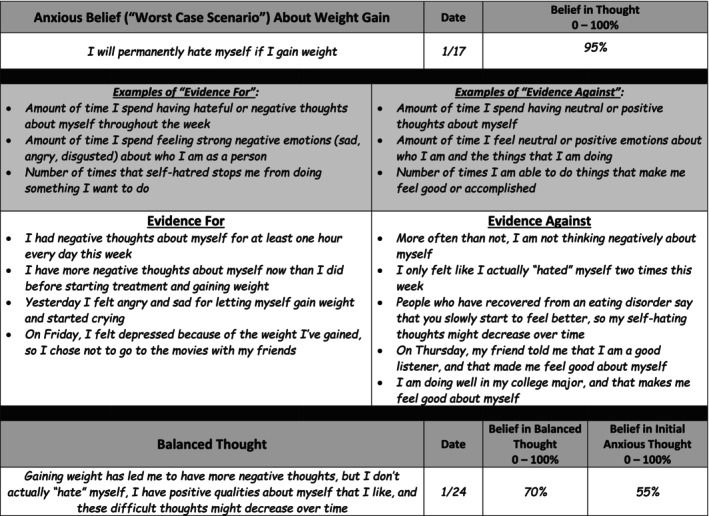
A sample cognitive restructuring worksheet targeting anxious beliefs about the consequences of weight gain.

We posit that cognitive restructuring can modify anxious beliefs about the consequences of weight gain identified during open weighing. By helping patients compare catastrophic predictions to their actual experiences during weight restoration, this approach transforms weight restoration into a behavioral experiment. After identifying catastrophic beliefs, therapists and patients could collaboratively develop criteria for evidence that would support and challenge these beliefs (see Figure 1). Taking an empirical stance, patients would frame their beliefs as hypotheses to test (Tolin 2024). Between sessions, patients could observe and record daily experiences that support or challenge their beliefs about weight gain consequences. In subsequent sessions, therapists and patients would review evidence and formulate balanced perspectives, aiming to reduce the intensity of beliefs. Questions and strategies from cognitive dissonance‐based (Stice et al. 2019), self‐compassion (Braun et al. 2016), and body positivity (Guest et al. 2019) interventions that counter thinness ideals and weight stigma may further assist patients in challenging negative beliefs about the consequences of weight gain.

The inhibitory learning approach cautions against pre‐exposure cognitive restructuring, which might dilute disconfirmation of feared expectations (Craske et al. 2014). Since both in‐session weighing and ongoing weight restoration could be framed as “exposure,” the cognitive restructuring interventions recommended here are typically conducted at the start of sessions, after the weekly “exposure” of living at a higher weight and before in‐session weighing. Further research is needed to determine optimal ways to pair cognitive restructuring with open weighing.

### 3.3 Combining Open Weighing With Imaginal Exposure

Imaginal exposure offers flexibility in targeting diverse weight‐related fears (Butler et al. 2024; Butler et al. 2023; Levinson et al. 2021, 2014). However, in vivo exposure typically produces superior outcomes for tangible stimuli (Foa et al. 1985; Gillihan et al. 2012), and research demonstrates that combining in vivo and imaginal exposure enhances treatment effectiveness for anxiety disorders (Abramowitz 1996; Abramowitz and Arch 2014). Therefore, integrating in vivo open weighing with imaginal exposure may optimize interventions targeting consequence‐related fears in EDs.

Imaginal exposure is theorized to work through several mechanisms: reducing the perceived credibility of catastrophic thoughts about weight gain; decreasing the emotional intensity of thoughts associated with weight gain consequences; and fostering a “bravery building” approach that enhances confidence in tolerating weight‐related cues (Butler et al. 2024; Butler and Heimberg 2023; Levinson et al. 2014). Combining imaginal exposure with open weighing may make imaginal exposure scripts feel more “real” and emotionally activating, potentially improving effectiveness.

In practice, patients could develop scripts describing feared “worst case scenarios.” Patients could then repeatedly read, listen to, or role‐play with the script while stepping on the scale. Therapists might incorporate elements like weighted vests to simulate higher weights and enhance emotional engagement (Schaumberg et al. 2021). These combined exposures should be designed collaboratively with patient input after ensuring patients understand the rationale.

## 4 Conclusion

Building on existing CBT protocols that address weight‐related concerns, we propose that open weighing sessions—when patients confront weight increases—provide underutilized therapeutic opportunities to work with consequence‐related beliefs in real time. The three enhancements presented here offer complementary techniques for systematically leveraging these emotionally activating moments to enhance CBT's effectiveness. Future research might include randomized controlled trials comparing different weighing approaches, mixed‐methods evaluations exploring weight‐related fears across ED diagnoses, clinical studies investigating the effectiveness of these recommended enhancements and optimal ways to combine cognitive restructuring with exposure therapy, and mechanistic research examining how addressing consequence‐related beliefs improves outcomes.

## Author Contributions

**Jamal H. Essayli:** conceptualization, writing – original draft, writing – review and editing. **Kaitlin A. Hill:** conceptualization, writing – review and editing. **Hana F. Zickgraf:** conceptualization, writing – review and editing. **Lauren N. Forrest:** conceptualization, writing – review and editing. **Cheri A. Levinson:** conceptualization, writing – review and editing.

## Conflicts of Interest

The authors declare no conflicts of interest.

## Data Availability

Data sharing not applicable to this article as no datasets were generated or analysed during the current study.
